# *Adesmia pinifolia,* a Native High-Andean Species, as a Potential Candidate for Phytoremediation of Cd and Hg

**DOI:** 10.3390/plants13040464

**Published:** 2024-02-06

**Authors:** Victoria Parera, M. Verónica Pérez-Chaca, Laura V. Gallardo, Camila V. Gatica-Aguilar, Carlos A. Parera, Gabriela E. Feresin

**Affiliations:** 1 Instituto de Biotecnología, Facultad de Ingeniería, Universidad Nacional de San Juan, Av. Libertador General San Martin 1109 Oeste, San Juan 5400, Argentina; gferesin@unsj.edu.ar; 2 Consejo Nacional de Investigaciones Científicas y Técnicas (CONICET), Godoy Cruz 2290, Cuidad Autónoma de Buenos Aires (CABA) C1425FQB, Argentina; caamigatica@gmail.com; 3 Facultad de Química, Bioquímica y Farmacia, Universidad Nacional de San Luis. Ejército de los Andes 950, San Luis 5700, Argentina; veroperezchaca@gmail.com (M.V.P.-C.); gallardolaurav@gmail.com (L.V.G.); 4 Instituto Nacional de Tecnología Agropecuaria (INTA), Avenida Rivadavia 1439, Cuidad Autónoma de Buenos Aires (CABA) C1033AAE, Argentina; parera.carlos@inta.gob.ar

**Keywords:** Fabaceae, native species, antioxidant defence system, heavy metals, oxidative stress, phytostabilisation

## Abstract

This study highlights *Adesmia pinifolia*, a native high-Andean species, as a potential candidate for the phytoremediation of soils contaminated with Cd and Hg. In this work, a semi-hydronic assay with different doses of Cd (3, 4.5, and 6 mg L^−1^) and Hg (0.8, 1.2, and 1.6 mg L^−1^) was analysed to evaluate the establishment of plants, antioxidant defence systems, oxidative stress, and the ability to accumulate heavy metals. The results indicate high survival rates (>80%); however, Cd significantly reduced shoot and root biomass, while Hg increased root biomass with the 1.6 mg L^−1^ treatment. Cd and Hg tend to accumulate more in roots (2534.24 µg/g and 596.4 µg g^−1^, respectively) compared to shoots (398.53 µg g^−1^ and 140.8 µg g^−1^, respectively). A significant decrease in the bioconcentration factor of Cd and Hg in roots was observed as metal levels increased, reaching the maximum value at 3 mg L^−1^ (805.59 ± 54.38) and 0.8 mg L^−1^ (804.54 ± 38.09). The translocation factor, <1 for both metals, suggests that translocation from roots to shoots is limited. An overproduction of reactive oxygen species (ROS) was observed, causing lipid peroxidation and oxidative damage to plant membranes. Tolerance strategies against subsequent toxicity indicate that enhanced glutathione reductase (GR) activity and glutathione (GSH) accumulation modulate Cd and Hg accumulation, toxicity, and tolerance.

## 1. Introduction

Mining production is an economic activity that significantly contributes to economic growth and development in many countries. Currently, along the Andes Mountains in Argentina (2500 and 5000 m above sea level), numerous mining operations are active for the extraction of gold and copper [1,2]. Conversely, mining activities are considered a significant source of heavy metal environmental pollution [3], as they produce large quantities of hazardous waste and mine tailings containing, principally, heavy metals like Cd, Cu, Ni, Pb, Hg, Zn, and As, which have a substantial impact on the ecosystem [4,5,6]. Unlike organic pollutants, heavy metals are non-degradable, continuously accumulating in the environment. This ongoing contamination causes long-term deleterious effects on the ecosystem and poses significant risks to both wildlife and human health [7,8]. Therefore, it is of great importance to find appropriate ways to minimise their impact on ecosystems. Phytoremediation is a plant-based environmental remediation technique that reduces, stabilises, or removes heavy metals from the environment [9,10]. Two phytoremediation techniques have been considered for the treatment of tailings and mining waste: phytoextraction and phytostabilisation [4]. Phytoextraction involves the extraction of metals from the soil by absorbing them in metal-accumulating plants [11]. Phytostabilisation aims to reduce metal mobility and bioavailability within the rhizosphere [12]. This can be achieved through metal precipitation, sorption, complexation, or physical adsorption induced by the plant itself, thus, preventing their dispersion through wind or water [13,14].

Plant selection is a critical step for the success of phytoremediation, as species vary widely in their ability to uptake or immobilise different contaminants [15,16]. The metal accumulation capacity of plants is also affected by their ability to survive oxidative damage caused by the production of reactive oxygen species (ROS) during exposure to toxic metals [17,18,19]. Plants have a complex enzymatic and non-enzymatic antioxidant defence system to maintain ROS levels compatible with normal cell function [20,21]. The main antioxidant enzymes that respond to heavy metal toxicity are catalase (CAT); the enzymes of the Foyer–Halliwell–Asada Cycle, ascorbate peroxidase (APX), monodehydroascorbate reductase (MDHAR), dehydroascorbate reductase (DHAR), and glutathione reductase (GR); as well as superoxide dismutase (SOD) and glutathione peroxidase (GPOX) [22]. Exposure to Hg and Cd induces changes in defence systems, regulating antioxidants to prevent damage caused by ROS production [23,24]. Therefore, for adequate plant selection, a comprehensive study of plant tolerance strategies is crucial to rehabilitate contaminated and degraded soils [25].

Species belonging to the Fabaceae family are especially suited to colonising soils contaminated or deprived of nutrients and immobilising toxic elements in the roots [26,27]. This ability is attributed to their deep root system and their capacity to form a symbiotic relationship with nitrogen-fixing rhizobacteria in the soil [28,29]. Furthermore, native species are also preferred because they are adapted to the environmental conditions of the area to be treated [30,31]. Among them, species of the genus Adesmia, endemic to arid and semi-arid high-Andean regions of Chile, Argentina, Bolivia, and Peru [32], have been reported as capable of accumulating Cu, Fe, Pb, and Zn originating from mine tailings [33,34]. In a previous study, *Adesmia pinifolia* and *A. subterranea,* native species of the Central Andes Mountains of Argentina, were able to germinate under different concentrations of heavy metals (Cd, Hg, Ni, and As). No toxic effects were observed on seed germination and early seedling growth in both species under different concentrations of Cd and Hg; hence, *Adesmia* species could be considered for remediating soils contaminated with Hg and Cd [35]. There are no studies available on the tolerance and ability of these species to accumulate heavy metals. The aim of this study was to evaluate plant establishment, antioxidant enzyme activity, and oxidative stress parameters in *A. pinifolia* under stress conditions induced by Cd and Hg. Additionally, this study aimed to determine the plant’s capacity to accumulate these heavy metals, providing insights into its phytoremediation potential.

## 2. Results

### 2.1. Survival and Growth

After 60 days of exposure to the treatments, the survival of *A. pinifolia* was recorded. The results showed that survival was more than 80% in all the treatments involving heavy metals and the control. There were no significant differences among the survival rates of the Cd treatments (*p* = 0.3933) and the Hg treatments (*p* = 0.8731) compared to the control (Table 1 and Table 2).

The growth behaviour of *A. pinifolia* was determined by measuring the shoot and root dry biomass. The effect of Cd concentration on growth depends on the type of organ (root or shoot) (two-way ANOVA, *p* < 0.0001). There was a significant decrease (*p* < 0.0001) in shoot biomass with increasing Cd concentrations (Table 1). In the 3 mg L^−1^, 4.5 mg L^−1^, and 6 mg L^−1^ Cd treatments, the shoot biomass decreased by 58.6%, 68.8%, and 74.8%, respectively, compared to that of the control. Root biomass was significantly reduced (*p* < 0.0001) by 90% under the Cd treatments compared to the control, but no differences between the treatments were observed (Table 1).

Similarly, as observed with the Cd treatments, the effect of Hg concentration on growth depends on the type of organ (root or shoot biomass) (two-way ANOVA, *p* < 0.0001). The Hg treatments significantly reduced the shoot biomass (*p* = 0.0052) compared to the control; the most significant reduction was observed at 1.2 mg L^−1^ Hg (−16.3%) (Table 2). The root biomass exhibited a significant reduction in the 0.8 and 1.2 mg L^−1^ treatments; however, at the highest Hg concentration, the root biomass increased by 21% compared to the control and by more than 200% compared to the other concentrations (Table 2).

### 2.2. Heavy Metal Uptake and Accumulation

The accumulation of Cd and Hg in shoots and roots after 60 days of exposure to the Cd treatments is presented in Table 3 and Table 4. The effect of different doses of Cd on root accumulation was more pronounced than that on the shoots of *A. pinifolia* (*p* < 0.0001), with values of 2534.24 µg g^−1^ and 398.53 µg g^−1^, respectively. In shoot tissues, the accumulation of Cd did not increase with rising amounts of spiked metals in the solution. The same tendency was observed for root tissue (Table 3).

The recorded BCF and TF values for *A. pinifolia* under different Cd concentrations are presented in Table 3. Furthermore, a two-way analysis of variance showed a significant effect of type of organ, Cd treatment, and the interaction between type of organ and Cd treatment on the bioconcentration factor. The results showed that all plant tissues (roots and shoots) had BCF values > 1 for all the applied levels of Cd. Nevertheless, roots showed significantly higher BCF values compared to shoots (*p* < 0.0001). The highest BCF of Cd in roots (805.59) was observed at a concentration of 3 mg L^−1^, six times higher than that of the shoot tissues (125.57). The BCF factor in roots significantly decreased as Cd levels increased (Table 3). However, the BCF values in shoot tissues were similar between treatments. All the TF values of Cd were less than 1.0 (Table 3) across all treatments, ranging from 0.13 (4.5 mg L^−1^) and 0.16 (3 mg L^−1^) to 0.19 (6 mg L^−1^), and indicating a low Cd translocation from roots to shoots.

The Hg content in *A. pinifolia* was significantly affected by Hg concentration, type of organ, and their interaction (*p* < 0.0173) (Table 4). The accumulation of Hg in roots showed a significant difference (*p* < 0.0048) among the applied levels of Hg, with the highest accumulation observed at 0.8 and 1.6 mg L^−1^ Hg (643.63 µg g^−1^ and 639.53 µg g^−1^, respectively). In shoots, no significant differences were observed among treatments (Table 4). The BCF was significantly affected by Hg concentration, type of organ, and their interaction (*p* < 0.0001). The BCF of roots decreased with the increasing concentration of Hg, being significantly higher under the treatment with 0.8 mg L^−1^ (804.54). A similar tendency was observed for the shoots, where a higher bioaccumulation value was detected when they were exposed to the Hg treatments at 0.8 mg L^−1^ (173.43). In all Hg treatments, TF values were less than 1.0, not evidencing significant differences between treatments and indicating a low Hg translocation from roots to shoots (Table 4).

### 2.3. Lipid Peroxidation and H_2_O_2_

**Figure 1:**
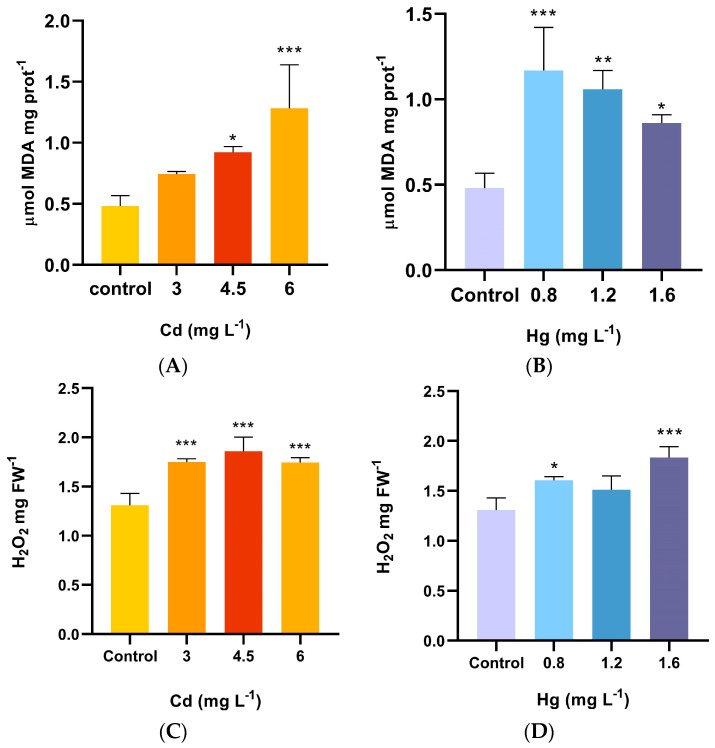
Changes in the (**A**,**B**) MDA and (**C**,**D**) H_2_O_2_ contents of shoots of *A. pinifolia* after 60 days of Cd (3, 4.5, and 6 mg L^−1^) or Hg (0.8, 1.2, and 1.6 mg L^1^) treatment. All values are means ± SD (*n* = 3). Significant differences at *** *p* < 0.001, ** *p* < 0.01, and * *p* < 0.05 as compared to the control (Tukey’s post hoc test); ns: not significant.

The results for MDA, an indicator of lipid peroxidation of the membrane, showed that the MDA concentrations of *A. pinifolia* were significantly increased (*p* < 0.001) compared to the control after the Cd treatments (Figure 1A). The MDA content rose with the increasing concentration of Cd, with the magnitude of elevation ranging from 1.8 to 2.2 folds at 3 mg L^−1^–6 mg L^−1^ Cd compared to the control. Mercury significantly induced the production of MDA in *A. pinifolia* compared to the control (*p* < 0.001). At 0.8 mg L^−1^ Hg, the MDA content reached its peak value at 1.17 µM MDAmg protein^−1^ (Figure 1B).

The plants growing in Cd treatments significantly enhanced (between 1.3 to 1.4 folds) H_2_O_2_ concentrations compared to the control (*p* < 0.0001) (Figure 1C). Under the Hg treatments, the same trend was observed (*p* < 0.0001) (Figure 1D).

### 2.4. Antioxidant Enzyme Activities and GSH

**Figure 2:**
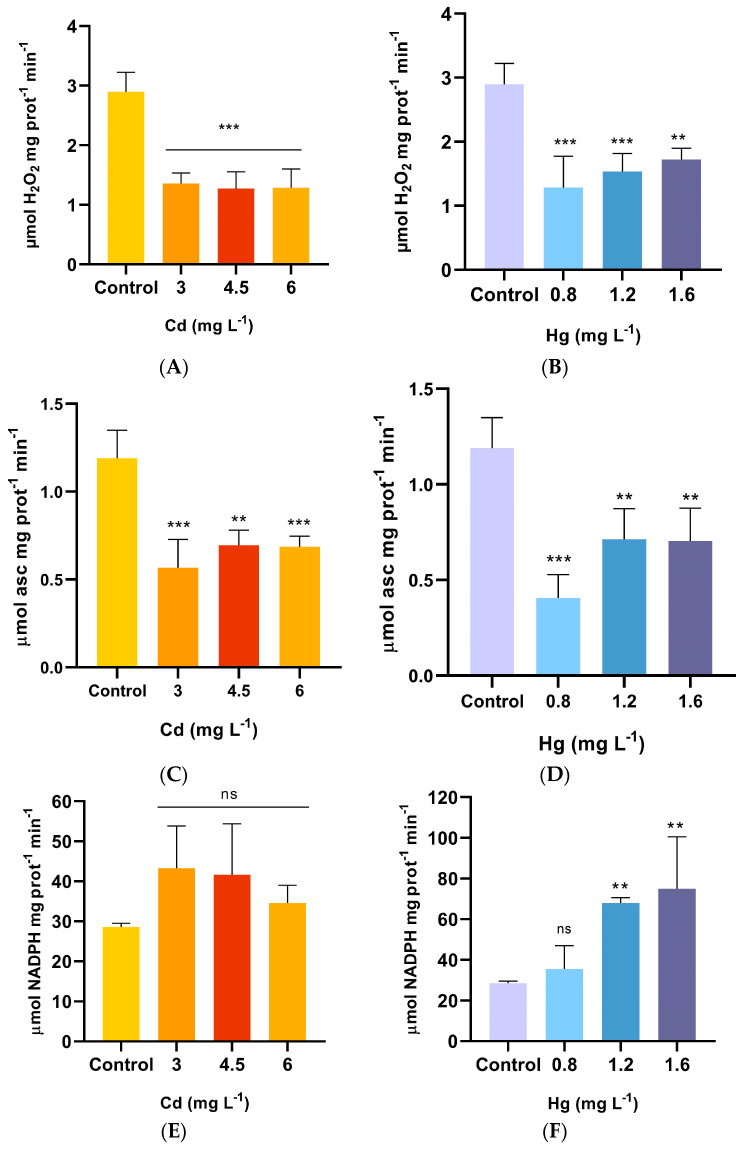
Effect of Cd and Hg on activity of (**A**,**B**) catalase (CAT), (**C**,**D**) ascorbate peroxidase (APX), and (**E**,**F**) glutathione reductase (GR) contents in shoots of *A. pinifolia* after 60 days of Cd (3, 4.5, and 6 mg L^−1^) or Hg (0.8, 1.2, and 1.6 mg L^−1^) treatment. All values are means ± SD (*n* = 3). Significant differences at *** *p* < 0.001, and ** *p* < 0.01 as compared to the control (Tukey’s post hoc test); ns: not significant.

Under the Cd treatments, CAT activity was significantly reduced (*p* < 0.0002) as compared to the control, exhibiting a general trend of decline as the Cd concentration increased (Figure 2A). All CAT activity values in the Hg treatments were lower than those in the control, and the activity showed a general trend to increase as the Hg-treated solution concentration rose, reaching its peak value at 1.6 mg L^−1^ (2.32 µmol H_2_O_2_mg protein^−1^ min^−1^) (Figure 2B).

Significant differences in APX activities were found between the Cd treatments and the control after 60 days exposure (*p* = 0.0002) (Figure 2C). APX activity was significantly inhibited at the rate of 43–31% compared to the control. There was a slight increase in APX activity as the Cd concentration increased. Regarding the Hg treatments, APX activity was significantly affected (*p* = 0.0001). In the lower Hg concentration treatment, APX was reduced by 60% compared to the control and then increased in the remaining treatments but was still significantly lower than in the control (Figure 2D).

GR activity in *A. pinifolia* treated with Cd showed no significant modification compared to the control (*p* = 0.1100) (Figure 2E), although a slight increase was noted with the 3 mg L^−1^ treatment. On the contrary, GR activity under the Hg treatments started to increase significantly (*p* = 0.0006) at 1.2 mg L^−1^ Hg and reached maximal values at 1.6 mg L^−1^ Hg, but no significant change was observed at 0.8 mg L^−1^ Hg compared to the control (Figure 2F).

**Figure 3:**
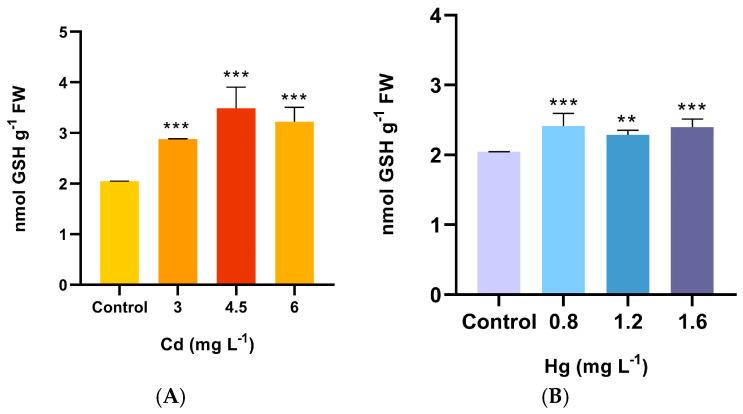
Glutathione (GSH) content in shoots of *A. pinifolia* aerial parts after 60 days of (**A**) Cd (3, 4.5, and 6 mg L^−1^) or (**B**) Hg (0.8, 1.2, and 1.6 mg L^−1^) treatment. All values are means ± SD (*n* = 3). Significant differences at *** *p* < 0.001 and ** *p* < 0.01 as compared to the control (Tukey’s post hoc test); ns: not significant.

Cadmium and mercury significantly increased the GSH content (*p* < 0.0001) in plants of *A. pinifolia* compared to untreated plants (Figure 3A,B). In the Cd treatments, the increase in GSH was more than 40%, and no differences among the treatments were observed. The same trend was detected for the Hg treatments, although similar GSH activity was detected between the treatments (Figure 3C).

## 3. Discussion

Cadmium is one of the Earth’s most toxic heavy metals, exerting negative effects on the growth and development of plants even at low concentrations [36]. The reduction in plant growth may result from the inhibitory effect of Cd on the synthesis of photosynthetic pigments, negatively impacting photosynthesis processes [37], or its interfering with the uptake of essential nutrients for growth and development [38].

One of the most important effects of Cd stress on legume plants is the inhibition or retardation of plant growth [39]. The results of this study show that *A. pinifolia* can survive and grow in the presence of Cd in the medium. This species is capable of surviving and developing even at the highest concentration evaluated (6 mg L^−1^). However, plant growth (shoot and root biomass) was severely reduced in the presence of this metal. When the concentration of Cd increased, the shoot biomass was lower, but the root dry weight remained unchanged. These results agree with other studies that have reported an inhibition of plant development and biomass decrease with higher concentrations of Cd in growth media for *Koelreuteria paniculata* [40], *Salix matsudana* and *Salix psammophila* [41], and *Chenopodium quinoa* [42]. This phenomenon may be attributed to the suppression of the cell division process, particularly in G1/S cells, as they are the most vulnerable to Cd treatment [43,44], and an increase in lignin content, which can limit cell expansion and nutrient uptake [45].

Despite its non-essential physiological role, Cd can be taken up by plants. According to Igbal et al. [46], plants absorb Cd, and it has the potential to be stored in the root tissues or transferred to the shoot tissues. Nevertheless, in the majority of plant species, cadmium tends to accumulate in the roots. In the present study, Cd accumulation was distinctly higher in roots compared to shoots, but the accumulation did not linearly increase with Cd concentration. Similar results were reported by Jawad Hassan et al. [47] in two sorghum cultivars (*Sorghum bicolor*) and other species, such as *Sesuvium portulacastrum* [48] and *Satureja hortensis* [49]. These results indicate that *A. pinifolia* has a strong ability to accumulate Cd in root tissue. This might be because roots are the first plant organs that come into contact with heavy metals that contaminate the soil. Roots can limit the translocation of these elements to the aboveground parts of the plant, sequestering and inactivating the metals, thereby stabilising their toxicity [50].

The uptake and accumulation ability of plant species can be assessed by analysing the bioconcentration and translocation factors based on the available metal fractions [51]. In this study, the BCF values in root and shoot tissues were higher than 1. However, BCF was significantly higher in the roots than the shoots and decreased with increasing Cd levels. These results indicate that this species has a strong capacity for Cd accumulation in its tissues. A large portion of Cd was stored in the roots, especially when the concentration in the solution was low, indicating that, when subjected to higher stress conditions, this species opted for an exclusion strategy over accumulation to prevent cell damage [52]. These results agree with the findings reported by Gishini et al. [53] and Ramana et al. [54] in their studies on Cd absorption and accumulation in *Prosopis farcta* (Fabaceae) and *Agave americana* (Asparagaceae), respectively, showing that, initially, Cd was accumulated in the roots and that some was translocated to aboveground parts. A translocation factor (TF) <1 is an important feature of plants well-suited for phytostabilisation [55], as it determines the metal transfer from roots to shoots. In this study, the TF was <1 for all the Cd treatments, confirming the ability of *A. pinifolia* to accumulate Cd in the root system, preventing significant Cd movement to the aboveground parts. 

Cadmium toxicity leads to an excessive generation of ROS (H_2_O_2_, O_2_^−^, and OH^−^), causing damage to plant membranes, oxidation of biomolecules, and destruction of cell organelles [56]. Therefore, the levels of H_2_O_2_ and MDA in plant tissues are considered important indices for evaluating the degree of antioxidant damage under heavy metal stress. Several investigations have demonstrated that the addition of Cd to growth media results in elevated levels of H_2_O_2_ and MDA accumulation [57,58]. In this study, exposure of *A. pinifolia* to Cd resulted in an overproduction of H_2_O_2_, between 1.3-to-1.4-fold relative to the control, leading to an increase in lipid peroxidation reflected in an elevation in MDA content. 

The plant defends itself against oxidative damage through a complex antioxidant system; however, the activities of antioxidant enzymes and the levels of non-enzymatic antioxidants in plants exposed to heavy metal stress can either be enhanced, remain unaffected, or be suppressed. These outcomes depend on factors such as plant species, types of metal ions, their concentrations, and exposure duration [59]. The results of the present study showed that CAT and APX activities were significantly inhibited by all Cd concentrations tested. The decrease in CAT and APX activity has been associated with Cd toxicity in *Triticum aestivum* seedlings [60], *Canna orchioides* leaves [61], and *Zea mays* [62]. The decrease in CAT activity might result from Cd-induced iron substitution in catalase active sites and insufficient iron absorption from the medium, or it may be that Cd leads to enzyme inhibition by interacting with thiol groups in their structure [63].

GSH activates antioxidant enzymes, such as superoxide dismutase (SOD) and Glutathione reductase (GR), which neutralise Cd-induced ROS to improve cellular redox homeostasis [64]. In our model, there is a significant increase in GSH, generating a tendency to stimulate GR activity in plants subjected to Cd stress. This leads to the assumption that the non-enzymatic pathway plays a crucial role in defending against contamination by Cd at its different concentrations. There are cellular characteristics that facilitate the life of metallophytes, associated with the efficiency of the antioxidant defence system that faces oxidative stress, maintaining cellular redox homeostasis through the synchronous action of several enzymes (SOD, CAT, POD, APX, and GR) and non-enzymatic antioxidants (AsA and GSH), as well as ROS transformation pathways. These mechanisms form the basis for adaptation to excessive amounts of heavy metals [65].

Mercury is a very toxic non-essential metal in plants, even at low concentrations [66]. Nevertheless, *A. pinifolia* was able to survive and thrive in the presence of various Hg concentrations, with over an 85% survival rate. Growth inhibition and a decrease in biomass production are commonly observed phenomena in plants due to the toxicity of Hg [67,68,69]. On the contrary, there are some plants species that have been able to grow more vigorously and healthily under certain Hg concentrations. Pérez-Sanz et al. [70] reported no inhibition and even some stimulation of the growth of *Silene vulgaris* exposed to 0.6 mg and 5.5 Hg kg^−1^ soil. Similar results were reported for *B. juncea*, *B. napus*, *A. codonocarpa*, and *T. Triandra* [71]. These results align with our findings, showing that under the treatment of 1.6 mg L^−1^, root growth was stimulated. In plant development, the absence of negative impacts caused by Hg can be attributed to a hormetic effect. Plants frequently undergo hormetic growth stimulation when exposed to low concentrations of toxic and non-essential metal ions [72,73]. Small amounts of mercury in plant cells could induce ROS, acting as signal molecules to improve the antioxidant response [23].

Plants exhibit variations in their capacity to absorb Hg, with the translocation of this liquid metal from the roots to the shoots of plants typically being limited [74]. Several studies have reported that concentrations of accumulated mercury are higher in roots than in the aboveground parts of *Marrubium vulgare* [75], *Sesbania grandiflora* [76], *Paspalum distichum* [77], and *Opuntia stricta*, *Aloe vera*, *Setcreasea purpurea*, *Chlorophytum comosum,* and *Oxalis corniculate* [78]. The data presented here agree with these findings, as *A. pinifolia* exhibited a strong capacity to accumulate Hg in roots, especially at the lowest Hg concentration treated (643.63 µg g^−1^). This may be attributed to the rapid absorption of heavy metals by roots, in contrast to the slower transportation of Hg to other plant tissues [74]. A BCF > 1 suggests a significant accumulation of heavy metals in biomass. The results obtained in this work revealed a BCF higher than 1 for roots and shoots. In the 0.8 mg L^−1^ Hg treatment, the BCFs were enhanced compared to the other treatments evaluated. This could imply a saturation event at low Hg concentrations, beyond which the plant might not be able to accumulate more mercury. Although there was a small amount of Hg translocating into the shoots, the TF was <1, probably indicating an exclusion strategy to avoid toxic effects on the aboveground parts, limiting its mobility once inside the plant [69].

A notable characteristic of the oxidative stress induced by Hg is the overproduction of reactive oxygen species (ROS) [79]. Lipid peroxidation caused by Hg stress has been reported in *Lemna minor* [80] and *Eichhornia crassipes* [81]. In this study, we observed an increase in the content of MDA and H_2_O_2_, causing damage mainly at the plasma membrane level, which indicates modifications in the redox state of the plant. Exposure to this pollutant also affected the activities of antioxidant enzymes differently. Among the three antioxidant enzymes tested, CAT and APX activity decreased significantly compared to the control but increased slightly when the concentration exceeded 1.2 mg L^−1^ Hg in the treatment. This could be attributed to H_2_O_2_ acting as a signal molecule to activate defence enzymes, as published by Perez Chaca et al. [23]. Similar results were also observed in *Chlamydomonas reinhardtii* when Hg concentrations were increased up to 8 μM [82]. On the other hand, higher concentrations of Hg resulted in higher GR activity. Similar outcomes were reported for *Fagopyrum tataricum* [83]. GSH, a powerful non-enzymatic antioxidant known for removing heavy metals from cells by chelating them in the cytoplasm [84], demonstrated induced GSH concentration under Hg treatment conditions, which indicates an active participation in detoxifying ROS.

The findings of this study underscore the potential of *A. pinifolia* to stabilise areas contaminated with Cd and Hg. Moreover, the presence of these metals triggers the enzymatic and non-enzymatic antioxidant system, which helps alleviate stress and promotes plant survival and growth.

## 4. Materials and Methods

### 4.1. Plant Material and Growing Conditions

#### 4.1.1. Plant Material and Seed Collection

*Adesmia pinifolia* (Gillies ex Hook. and Arn) is a shrub that reaches a height of 1.5–2 m and is found at elevations between 1500 and 3700 m asl., along the Andes Mountains of San Juan, Mendoza, and Neuquén (Argentina), as well as Chile [32,85]. Seeds of *A. pinifolia* were randomly hand-collected from the Cordón del Plata Provincial Park (32°58′50.0″ S 69°20′58.6″ W). After harvesting, seeds were extracted from the fruit capsules and subsequently stored at 4 °C until they were utilised.

#### 4.1.2. Growing Conditions

Before use, the seeds were scarified in sulfuric acid (H_2_SO_4_) for 5 min and then washed several times with tap water to break seed dormancy [86]. Then, the seeds were sown in seedling trays in a semi-hydroponic system, using perlite as the substrate, and grown in a growth chamber under controlled environmental conditions for 60 days. The growth conditions were 23 ± 2 °C (constant) with a photoperiod of 12 h light/12 h dark (photon flux density of 1500 μmol m^−2^ s^−1^) and 60%–65% relative humidity. The test solutions (heavy metal treatments and nutrient solutions) replacement occurred every three days in order to maintain optimal nutrient availability and prevent nutrient depletion. The pH level was maintained at 5.5 to ensure stability. The Hoagland nutrient solution included: 75 mM KNO_3_, 32.5 mM Mg (NO_3_)2 6 H_2_O), 1 mM KH_2_PO_4_, 0.8 mM H_3_BO_3_, 0.2 mM MnSO_4_ H_2_O, 0.2 mM ZnSO_4_ 7 H_2_O, 0.2 mM CuSO_4_ 5 H_2_O, 0.2 mM (NH_4_)6 Mo7O_24_ 4 H_2_O, 10 mM SO_4_Ca ^1^/_2_ H_2_O.

### 4.2. Heavy Metal Treatments

Heavy metal solutions were prepared as: Cd (ClCd_2_.H_2_O) and Hg (Hg (NO_3_)_2_.H_2_O). For the experiments, three concentrations were considered for each metal: Cd (3, 4.5, and 6 mg L^−1^) and Hg (0.8, 1.2, and 1.6 mg L^−1^). Distilled water was used as the control (C). The pH of the heavy metal solutions was adjusted to 5.5 using HCl (2N). Each treatment consisted of three replicates (25 cell holes of 50 cc), with 25 plants per replicate. The plant tray distribution was randomised to avoid edge effects and fluctuations in the growth chamber. The concentrations of Cd and Hg selected for the heavy metal treatments represent the maximum concentrations allowed for these elements in soils for agricultural use according to the National Legislation of Argentina (Law No. 24.051 “Régimen de Desechos Peligrosos” Decreto 831/93) and 150% and 200% higher values.

### 4.3. Harvesting of Plant Materials

After 60 days of treatment, the survival rate and growth of plants was recorded. Then, the plants were harvested from the seedling trays and separated into shoots (leaves and stems) and roots. The plant samples were soaked and rinsed in deionised water several times, cleaned with tissue paper [87], and oven-dried at 70 °C for 48 h until no further weight change was observed. The final weight was recorded for biomass estimation. For enzymatic activities and MDA analysis, shoot samples were immediately immersed in liquid nitrogen, then stored in the freezer at 80 °C.

### 4.4. Hydrogen Peroxide (H_2_O_2_)

The H_2_O_2_ content was determined according to Alexieva et al. [88]. Shoot fresh samples (100 mg) were homogenized with 5 mL of TCA (0.1%) in liquid nitrogen and centrifuged at 12,000× *g* for 15 min. The supernatant was mixed with 0.5 mL of 10 mM phosphate buffer (pH 7.0) and 1 mL of 1 M potassium iodide. The absorbance at 390 nm was recorded using a UV-Vis spectrophotometer. The H_2_O_2_ content was determined using the extinction coefficient at 0.28 mM^−1^ cm^−1^ and was expressed as nmol g^−1^ FW.

### 4.5. Determination of MDA Concentration

In this assay, the MDA content was determined as follow: the shoot tissue was homogenised at 14,000 rpm for 15 min. The supernatant was homogenised with 20% trichloroacetic acid and 0.5% thiobarbituric acid in 20% trichloroacetic acid, in equal volumes. We incubated the homogenate at 95 °C for 15 min and then placed it on ice to cool for 5 min. Then, it was centrifugated at 10,000 rpm for 5 min to precipitate the proteins. The supernatant was used to measure the MDA content (e ε560 nm = 155 mM^−1^ cm^−1^) by measuring the absorbance at 532 nm and then at 600 nm to correct for nonspecific turbidity. The concentration of MDA was expressed as µmol mg^−1^ of Heath and Packer protein [89].

### 4.6. Enzymatic Activities

From extracts (100 mg) of shoot tissue, the activities of antioxidant enzymes were determined. The frozen samples were crushed in liquid N_2_ using a mortar and a 0.1 M Tris-HCl buffer (pH 8.0) to which 0.1 M ethylenediaminetetraacetic acid and 1 mM phenylmethanesulfonyl fluoride were added in a 1:2 ratio (p:v), and Triton X-100 was added at 0.2% (*v*/*v*). At 23,326× *g* for 30 min at 4 °C, the homogenate was centrifuged. The supernatants were taken to determine the enzymatic activities.

To determine CAT activity (EC 1.11.1.6), a reaction mixture containing 50 mM potassium phosphate buffer (pH 7.0) and 10.5 mM H_2_O_2_ was used. After adding the enzyme extract, the reaction was carried out at 25 °C for 2 min. The enzyme activity was measured by the rate of decrease in absorbance at 240 nm (ε = E = 39.4 mM^−1^ cm^−1^) that was evaluated spectrophotometrically. CAT activity was calculated by measuring the disappearance rate of H_2_O_2_ at 240 nm [90].

APX activity (EC 1.11.1.11) was measured through a reaction mixture consisting of 50 mM phosphate buffer (pH 7.4), 5 mM ascorbic acid, and enzyme extract [91]. The reaction was started by adding 100 µL of 0.1 mM H_2_O_2_, and the rate of H_2_O_2_-dependent oxidation of ascorbic acid was determined by following the decrease in absorbance at 290 nm for 2 min. Protein concentration was analysed using bovine serum albumin as a standard [92].

The GR enzymatic activity assay (EC 1.6.4.2) was performed by adding to the shoot sample a reaction mixture containing 1 mL of assay buffer (50 mM phosphate buffer containing 2 mM EDTA (pH 7.5), 20 mM GSSG, 2 mM NADPH, and 20 µL of protein sample. GR activity was measured by the decrease in absorbance at 340 nm per minute for a total period of 2 min, determining the oxidation of NADPH per minute. The extinction coefficient of GR was 6.2 mM^−1^ cm^−1^. The total protein concentration was calculated using the Bradford method. GR activity was expressed in µmol NADPH mg protein^−1^ min^−1^ [93].

### 4.7. Non-Enzymatic Antioxidants

To determine GSH, the fresh shoot tissue (100 mg) was homogenised with 1 mL of 0.1 N HCl (pH 2), 1% PVP (*w*/*v*), which for 20 min at 4000× *g*, 4 °C, was centrifuged. GSH content was estimated by incubating 100 µL of sample with phosphate buffer (0.5 M H_2_PO_4_/K_2_HPO_4_, containing 0.1 g of ascorbic acid and 0.05 g of glyoxylic acid (pH 6.8), for 5 min at 60°, after which time it was quickly cooled on ice. Ellman’s reagent (DTNB solution, 0.100 mL) was added to all tubes, mixed, and then centrifuged at 12,000 rpm for 2 min. The GSH content was measured at Abs 412 nm. A control tube containing only buffer and GSH standard was used from a 4 mM GSH stock solution and diluted 1:10. The results were expressed as nmol of GSH g^−1^ FW [94].

### 4.8. Quantification of Cd and Hg in Plant Tissues

#### 4.8.1. Total Cd and Hg Concentration

The total Cd content in plant tissues (roots and shoots) was determined by taking 0.1–3 g of the plant material and drying it in an oven until the sample was thoroughly dry. Then, the sample was placed in a cool muffle furnace (450 ± 20 °C). Then, 1 mL of concentrated HNO_3_ was added to the ash, dried, and returned to the muffle furnace. Finally, the ash was dissolved in 5–10 mL of 1 N HCl. Concentrations of Cd were determined by an atomic absorption spectrometer (Shimadzu AA-7000) equipped with a graphite furnace and an ASC-7000 autosampler.

For Hg determination, a wet digestion of the shoot and root samples was carried out. Plant material (1 ± 0.05 g) was placed in a Teflon vessel, then 5 mL of redistilled water and 5 mL of concentrated (65%) HNO_3_ were added. Wet digestion was performed in a hot bath (65 °C) for 24 h. Subsequently, the solution was diluted using redistilled water (up to 25 mL) in a volumetric flask. Mercury concentration was quantified using the cold vapour technique, specifically, cold vapour atomic absorption spectrometry (CVAAS), with the Shimadzu 7000-AA spectrometer (Tokyo, Japan).

#### 4.8.2. Calculation of Bioconcentration Factor and Translocation Factor

To assess the absorption ability of *A. pinifolia* for Cd and Hg, both the bioconcentration factor (BCF) and the translocation factor (TF) were calculated for each organ (shoots and roots) as follows [95]:

$$BCF=\frac{\left[metal\right]\;shoots\;or\;roots}{\left[metal\right]\;solution}$$

$$TF=\frac{\left[metal\right]\;shoots}{\left[metal\right]\;roots}$$

### 4.9. Data and Statistical Analyses

Two-way ANOVA (biomass, heavy metal concentration, and BCF) or one-way ANOVA (survival and antioxidant parameters), followed by Tukey’s multiple comparisons test (probability levels of 0.05, 0.01, and 0.001), was performed using GraphPad Prism version 8.0.0 for Windows.

## 5. Conclusions

The results of this study highlight for the first time the remarkable resilience of *Adesmia pinifolia*, with survival rates exceeding 80% across all the Cd and Hg treatments, even at high concentrations. When exposed to the Cd treatments, the plant exhibited the following responses: induced growth inhibition with increasing concentrations, which resulted in the overproduction of ROS, causing lipid peroxidation and oxidative damage to plant membranes, and increased GSH accumulation, indicating active involvement in chelating Cd and mitigating the effects of oxidative stress. In the presence of Hg, the plant exhibited: induced root growth at 1.6 mg L^−1^, with slightly reduced aerial part growth but with significant accumulation of intracellular H_2_O_2_, potentially acting as a signal molecule, activating the antioxidant defence system, and increased activities of GR antioxidant enzymes and accumulation of GSH, as main defence molecules. Concerning the BCF (>1) and TF (<1) values, *Adesmia pinifolia* can be considered as a potential candidate for phytoremediation of soils contaminated with Cd and Hg, particularly for phytostabilisation, due to its strong root-based Cd and Hg accumulation, especially at lower concentrations. The findings of the current experiment warrant further studies to open the possibility for more research work to be undertaken. These studies will verify the assumption of the phytoremediation potential of this species, considering higher concentrations of Cd and Hg, and the evaluation of the behavior under soil conditions.

## Figures and Tables

**Table 1:** Survival and growth (dry biomass) of *A. pinifolia* exposed to Cd treatments (3, 4.5, and 6 mg L^−1^) and control (distilled water).

Heavy Metal Treatment (mg L^−1^)		Survival (%)	Shoot Biomass (mg)	Root Biomass (mg)
Control		92 ± 8 a	29.69 ± 1.11 a	15.87 ± 1.41 a
Cd	3	85.3 ± 4.6 a	11.2 ± 1.41 b	1.51 ± 0.38 b
	4.5	81.3 ± 8.3 a	8.67 ± 1.28 c	1.45 ± 0.36 b
	6	84 ± 8 a	6.93 ± 0.93 d	1.45 ± 0.35 b

Note: Data represent the averages of six plants. Values are expressed as means ± SD. Different letters denote significant differences between treatments (*p* < 0.05) according to Tukey’s post hoc test.

**Table 2:** Survival and growth (dry biomass) of *A. pinifolia* exposed to Hg treatments (0.8, 1.2, and 1.6 mg L^−1^) and control (distilled water).

Heavy Metal Treatment (mg L^−1^)		Survival (%)	Shoot Biomass (mg)	Root Biomass (mg)
Control		92 ± 8 a	29.69 ± 1.11 a	15.87 ± 1.41 a
Hg	0.8	88 ± 8 a	25.93 ± 3.56 ab	7.94 ± 2.67 b
	1.2	90.6 ± 2.3 a	23.99 ± 3.28 b	6.78 ± 2.42 b
	1.6	85.3 ± 12.8 a	27.09 ± 5.3 ab	18.2 ± 4.73 a

Note: Data represent the averages of six plants. Values are expressed as means ± SD. Different letters denote significant differences between treatments (*p* < 0.05) according to Tukey’s post hoc test.

**Table 3:** Cd concentration in plant roots and shoots (µg g^−1^) and BCF and TF values of *A. pinifolia* plants treated with Cd (3, 4.5, and 6 mg L^−1^) and control.

Heavy Metal Treatment (mg L^−1^)		Metal Concentration (µg g^−1^)		BCF		TF
		Shoots	Roots	Shoots	Roots	
Control		ND	ND	-	-	-
Cd	3	302.97 ± 18.04 a	2416.76 ± 30.47 b	125.57 ± 48.06 a	805.59 ± 54.38 a	0.12 ± 0.03 a
	4.5	346.56 ± 14.18 a	2605.24 ± 29.47 b	77.01 ± 23.85 a	578.94 ± 94.96 b	0.13 ± 0.04 a
	6	546.06 ± 39.96 a	2580.10 ± 6.72 b	91.01 ± 14.74 a	475.34 ± 85.82 c	0.19 ± 0.04 a

Note: Statistically significant differences between means are marked with different letters at *p* < 0.05 (Tukey’s post hoc test).

**Table 4:** Hg concentration in plant roots and shoots (µg g^−1^) and BCF and TF values of *A. pinifolia* plants treated with Hg (0.8, 1.2, and 1.6 mg L^−1^) and control.

Heavy Metal Treatment (mg L^−1^)		Metal Concentration (µg g^−1^)		BCF		TF
		Shoots	Roots	Shoots	Roots	
Control		ND	ND	-	-	-
Hg	0.8	138.74 ± 16.25 a	643.63 ± 30.47 a	173.43 ±20.30 a	804.54 ± 38.09 a	0.22 ± 0.02 a
	1.2	118.75 ± 14.18 a	506.11 ± 52.34 b	98.96 ± 11.82 b	421.76 ± 43.62 b	0.24 ± 0.04 a
	1.6	164.91 ± 39.96 a	639.53 ± 6.72 a	103.07 ± 24.98 b	399.71 ± 4.20 b	0.26 ± 0.06 a

Note: Statistically significant differences between means are marked with different letters at *p* < 0.05 (Tukey’s post hoc test).

## Data Availability

Data are contained within the article.
